# Retraction Note: The traditional tibetan medicine *Yukyung Karne* exhibits a potent anti-metastatic activity by inhibiting the epithelial to mesenchymal transition and cell migration

**DOI:** 10.1186/s12906-023-03918-9

**Published:** 2023-03-22

**Authors:** Tenzin Choedon, Ganeshan Mathan, Vijay Kumar

**Affiliations:** 1 Virology Group, International Centre for Genetic Engineering and Biotechnology, Aruna Asaf Ali Marg, 110067 New Delhi, India; 2 Department of Biomedical Science, Bharathidasan University, 620024 Tiruchirappalli, India

**Retraction Note: BMC Complement Med Ther 15, 182 (2015)**

10.1186/s12906-015-0707-3

The Editor has retracted this article. After publication, concerns were raised regarding similar western blot control images in this article and the authors’ previous publication [1]. The authors explained that the data in the two articles originated from the same experiments. Further checks by the Publisher identified the use of the SKOV6 cell line, which is reported to be contaminated with HeLa cells, making it an unsuitable model for ovarian cancer. This undermines the conclusion that *Yukyung Karne* could be used in the management of ovarian cancer metastasis. Due to the cell line issue, the Editor no longer has confidence in the conclusions of this article.

None of the authors agree to this retraction.

## References

[1] Choedon T, Dolma D, Mathan G (2014). Molecular insights into the anti-cancer properties of traditional tibetan medicine Yukyung Karne. BMC Complement Altern Med.

